# High likelihood of actionable pathogenic variant detection in breast cancer genes in women with very early onset breast cancer

**DOI:** 10.1136/jmedgenet-2020-107347

**Published:** 2021-03-23

**Authors:** D Gareth Evans, Elke Maria van Veen, Helen J Byers, Sarah J Evans, George J Burghel, Emma Roisin Woodward, Elaine F Harkness, Diana M Eccles, Stephanie L Greville-Haygate, Jamie M Ellingford, Naomi L Bowers, Marta Pereira, Andrew J Wallace, Sasha J Howell, Anthony Howell, Fiona Lalloo, William G Newman, Miriam Jane Smith

**Affiliations:** 1 Manchester Centre for Genomic Medicine, St Mary’s Hospital, Manchester University Hospitals NHS Foundation Trust, Manchester Academic Health Science Centre, Manchester, UK; 2 Division of Evolution and Genomic Sciences, School of Biological Sciences, Faculty of Biology, Medicine and Health, Manchester Academic Health Science Centre, University of Manchester, Manchester, UK; 3 Prevent Breast Cancer Centre, Wythenshawe Hospital Manchester, University NHS Foundation Trust, Manchester, UK; 4 Department of Histopathology, Manchester University Hospitals NHS Foundation Trust, Manchester, UK; 5 Division of Informatics, Imaging and Data Sciences, School of Health Sciences, Faculty of Biology, Medicine and Health, University of Manchester, Manchester, UK; 6 University of Southampton and University Hospital Southampton, Southampton, UK; 7 Division of Cancer Sciences, Faculty of Biology, Medicine and Health, Manchester Academic Health Science Centre, University of Manchester, Manchester, UK

**Keywords:** genetics, genetic testing, human genetics

## Abstract

**Background:**

While the likelihood of identifying constitutional breast cancer-associated *BRCA1*, *BRCA2* and *TP53* pathogenic variants (PVs) increases with earlier diagnosis age, little is known about the correlation with age at diagnosis in other predisposition genes. Here, we assessed the contribution of known breast cancer-associated genes to very early onset disease.

**Methods:**

Sequencing of *BRCA1*, *BRCA2, TP53* and *CHEK2* c.1100delC was undertaken in women with breast cancer diagnosed ≤30 years. Those testing negative were screened for PVs in a minimum of eight additional breast cancer-associated genes. Rates of PVs were compared with cases ≤30 years from the Prospective study of Outcomes in Sporadic vs Hereditary breast cancer (POSH) study.

**Results:**

Testing 379 women with breast cancer aged ≤30 years identified 75 PVs (19.7%) in *BRCA1*, 35 (9.2%) in *BRCA2*, 22 (5.8%) in *TP53* and 2 (0.5%) *CHEK2* c.1100delC. Extended screening of 184 PV negative women only identified eight additional actionable PVs. *BRCA1/2* PVs were more common in women aged 26–30 years than in younger women (p=0.0083) although the younger age group had rates more similar to those in the POSH cohort. Out of 26 women with ductal carcinoma *in situ* (DCIS) alone, most were high-grade and 11/26 (42.3%) had a PV (*TP53*=6, *BRCA2*=2, *BRCA1*=2, *PALB2*=1). This PV yield is similar to the 61 (48.8%) *BRCA1/2* PVs identified in 125 women with triple-negative breast cancer. The POSH cohort specifically excluded pure DCIS which may explain lower *TP53* PV rates in this group (1.7%).

**Conclusion:**

The rates of *BRCA1*, *BRCA2* and *TP53* PVs are high in very early onset breast cancer, with limited benefit from testing of additional breast cancer-associated genes.

## Introduction

In recent years, a large increase in the use of multigene panel tests for breast cancer associated pathogenic variants (PVs) has expanded the number of potentially actionable PVs beyond *BRCA1* and *BRCA2*.^1–9^ These studies have shown an almost equal rate of *BRCA1/2* PVs to all additional potentially actionable gene PVs combined. In addition, much of the increased detection is due to variants in less actionable moderate-risk genes,^10^ *ATM* and *CHEK2*, with higher background population prevalence. The only other actionable breast cancer gene variants consistently identified at substantial rates is *PALB2*, which is now also considered to be a high-risk susceptibility gene.^11^

Although higher frequencies of actionable gene variants are reported in those at particularly young ages (<40 years) particularly for *TP53*, the PV rates of *ATM* and *CHEK2* do not appear to be strongly related if at all to age-at-onset, although a small effect was seen for *CHEK2* in two studies.^1 2^ Very few studies have concentrated testing on women with very early onset breast cancer. We previously reported a high rate of *BRCA1, BRCA2* and *TP53* PVs in a population based series of breast cancer in women ≤30 years of age at diagnosis.^12 13^ Fewer than 1 in 1000 women develop breast cancer by age 30 years and UK statistics showed that only 222 of 54 450 (0.41%) of breast cancers occurred in women aged <30 years^14^ (0.59% if ~100 breast cancers in women aged 30 years are included).^14^ Although this is a small group of patients with breast cancer, the prognosis of breast cancer diagnosed in this young age group is poor.^12 13 15 16^ *BRCA1* and *BRCA2* PVs have been reported in small numbers of women diagnosed aged ≤30 years; however, the studies reporting these individuals include many women with breast cancer diagnosed at older ages and do not specify the detection rates within the ≤30 years age group.^15 16^ The Prospective study of Outcomes in Sporadic vs Hereditary breast cancer (POSH) reported a 12% rate of *BRCA1/2* PVs in 338 of 2733 women diagnosed aged ≤40 years, but only 316 of a total 3095 women in POSH were aged ≤30 years and no separate analysis was presented.^15 16^ In another study, the rate of *TP53* PVs was reported as 6% in an unselected subset within 333 women with breast cancer aged ≤30 years.^17^ The Myriad study is the only large study that has assessed the detection rate of PVs in other breast cancer genes in women with breast cancer aged <30 years. In this study, 783 (2.2%) of 35 409 women were aged <30 years;^6^ however, it is likely that there was considerable pretesting in this series for *BRCA1/2* and *TP53* PVs as acknowledged by the authors and evidenced by the low detection rates among Ashkenazi Jews.

We present analysis of *BRCA1/2* and *TP53* testing in 379 patients with breast cancer aged ≤30 years, and of extended testing of a panel of additional breast cancer genes in 184 patients, expanding our previous population-based study of 115 women.^12 13^

## Methods

Individuals with a confirmed breast cancer diagnosis aged ≤30 years were eligible for the study. Affected women came from two sources. The first was a population-based study of 288 women with breast cancer presenting between January 1980 and December 1997 from the Manchester region (population=4.5M) of North-West England identified from the regional cancer registry.^12 13^ From this, 175 women were alive and potentially available for genetic testing.^12^ Fifty (28.6%) of these did not provide a DNA sample (it was either not appropriate to recontact or the individual did not wish to participate or could not be traced). This increases by 10 the number with available DNA samples from our previous report to 125.^13^ Only 39 currently living patients have not consented to the study. An additional 256 women were referred to the Manchester Centre for Genomic Medicine (MCGM) between 1990 and 2019. All women gave clinical consent for testing of breast cancer genes. Samples were initially screened for point mutations and copy number variants in *BRCA1, BRCA2*, *TP53* and for the *CHEK2* c.1100delC PV.^13^ When a PV was identified, no further testing was carried out. Samples testing negative were selected for next generation sequencing panels which included, as a minimum, the additional breast cancer associated genes: *PALB2*, *CHEK2*, *ATM*, *CDH1*, *PTEN*, *RAD50*, *RAD51D* and *NBN*. In addition, 1567 population control samples without breast cancer at entry aged 47–73 years from the PROCAS study^18^ were tested as part of the Breast Cancer Risk after Diagnostic Gene Sequencing (BRIDGES) programme.^19^

PV frequencies in the Manchester early onset cohort were compared with PV frequencies observed in women aged ≤30 years who took part in the prospectively ascertained POSH study (01/2000–01/2008).^15 16^

Tumour pathology information was obtained through hospital record and cancer registries. The pathology adjusted Manchester Scoring System was used to assess likelihoods of *BRCA1/2* PVs.^20^ Pathology-adjusted Manchester score (MS) of 15–19 is equivalent to a 10% probability of a *BRCA1/2* PV and a 20–24 point score is equivalent to a 20% probability.

The type and number of PVs were determined in the full cohort as well as in different age groups, specific tumour pathology characteristics and MS.

## Results

A total of 381 women with breast cancer diagnosed ≤30 years were included. Two women met diagnostic criteria for neurofibromatosis type 1, explaining their early onset of breast cancer. The remaining 379 were screened for variants in *BRCA1, BRCA2*, *TP53* and the *CHEK2* c.1100delC variant. This strategy detected 134 PVs: *BRCA1*=75 (19.79%), *BRCA2*=35 (9.23%), *TP53*=22 (5.80%), *CHEK2* c.1100delC=2 (0.53%). One woman harboured both a *BRCA1* and *BRCA2* PV. Of those testing negative, 184 (74.8%) underwent extended genetic testing. Sixty-two women did not undergo further testing due to poor quality, or insufficient, DNA. The detection rate was 4.35% (n=8) for actionable breast cancer PVs (*ATM*=2, *PALB2*=4, *CHEK2*=1, *PTEN*=1, online supplemental table 1). Single PVs were identified in other genes associated with breast cancer risk, *BRIP1* (c.2392C>T; p.Arg798Ter), *RECQL* (c.1667_1667+3delAGTA; p.?) and *RAD50* (c.1300_ 1306del; p.Asp434LysfsTer7).

10.1136/jmedgenet-2020-107347.supp1 Supplementary data

Risk associations for each gene were determined using the population controls from the PROCAS study (table 1). Significant associations with a more than twofold increased risk were found for *BRCA1*: OR=193.10 (95% CI 51.58 to 804.8), *BRCA2*: OR=17.61 (95% CI 8.59 to 36.53), *TP53*: OR=308.10 (95% CI 51.20 to 3202) and *PALB2*: OR=11.59 (95% CI 3.08 to 46.15). PV rates in the POSH study were established among the 287 women with invasive breast cancer at the age of ≤30 years. A total of 56 (19.5%) PVs were identified in *BRCA1* (32 PVs, 11.1%), *BRCA2* (17 PVs 5.9%), *TP53* (5 PVs, 1.7%) and *CHEK2* c.1100delC (3 PVs, 1.1%) (table 1).

**Table 1:** Association of pathogenic variants with early onset of breast cancer

	Total	*BRCA1*	*BRCA2*	TP53*	*CHEK2†*	*PALB2*	*ATM*	*BRIP1*	*RAD50*	*RECQL*
PROCAS controls	1567	2	9	0	6	3	6	2	6	5
%		0.13%	0.57%	0.00%	0.38%	0.19%	0.38%	0.13%	0.38%	0.32%
Breast cancer ≤30 study overall	379/184‡	75	35	22	2	4	2	1	1	1
%		19.79%	9.23%	5.80%	0.53%	2.17%	1.09%	0.54%	0.54%	0.54%
P value		**<0.0001**	**<0.0001**	**<0.0001**	0.6576	**0.0032**	0.1844	0.2847	0.5409	0.4868
Population based cohort	125/46	23	11	5	0	0	0	1	0	0
%		18.4%	8.8%	4.0%				2.17%		
P value		**<0.0001**	**<0.0001**	**<0.0001**				0.0832		
Referral to MCGM	254/138	52	24	17	2	4	2	0	1	1
%		20.47%	9.45%	6.69%	0.78%	2.90%	1.45%		0.72%	0.72%
P value		**<0.0001**	**<0.0001**	**<0.0001**	0.1320	**0.0012**	0.1320		0.4467	0.3978
POSH study	287	32	17	5	3					
%		11.15%	5.92%	1.74%	1.05%					
P value		**<0.0001**	**<0.0001**	**<0.0001**	0.1508					

*TP53 p value is based on population frequency of 1/5000.

†*CHEK2* p value is calculated for c.1100delC only.

‡Total of women tested for BRCA1/2, *TP53* variants and *CHEK2* c.1100delC is 379, total number of women tested for an extended panel of genes is 184.

MCGM, Manchester Centre for Genomic Medicine.

### Detection rate of pathogenic variants in different age groups

Surprisingly, the youngest age group (<26 years) showed a lower rate of *BRCA1/2* PVs; only 9/61 (14.75%) compared with 101/318 (31.76%) for those aged 26–30 years (p=0.0083) (table 2). *TP53* showed the reverse trend with 7/61 (11.48%) aged <26 years compared with 4.72% (15/318) in those aged 26–30 years (p=0.0649). Thus, only 12.93% (15/116) PVs in *BRCA1*/*2*/*TP53* in those aged 26–30 years were in *TP53* compared with 43.8% (7/16) in those <26 years (p=0.0060). The lower rates in the younger age group for *BRCA1/2* PVs were similar to the rates in the POSH cohort ≤30 years potentially reflecting ascertainment differences. The higher rate of *TP53* PVs (5.8%) compared with 1.7% in POSH likely reflects that the POSH study specifically excluded women with only DCIS and no invasive tumour component.

**Table 2:** Rates of pathogenic variants by age group, pathology and Manchester Scoring System

	Total cases	Total PVs	%	*BRCA1*	%	*BRCA2*	%	*TP53*	%	*BRCA1/BRCA2/* *TP53* combined	Other genes	Genes
Age <26	61	18	29.5%	6	9.8%	3	4.9%	7	11.5%	26.2%	2	*ATM; PALB2*
26–30*	318	127	39.9%	69	21.7%	32	10.1%	15	4.7%	36.5%	11	*ATM; BRIP1; CHEK2 (3); PALB2 (3); PTEN; RAD50; RECQL*
Total	379	145	38.3%	75	19.8%	35	9.2%	22	5.8%	34.8%	13	
Receptor status												
TNBC	125	61	48.8%	51	40.8%	6	4.8%	3	2.4%	48.0%	1	*PALB2*
HER2+	43	12	27.9%	1	2.3%	2	4.7%	8	18.6%	25.6%	1	*RECQL*
ER+/HER2-	79	30	38.0%	14	17.7%	8	10.1%	2	2.5%	30.4%	6	*ATM; CHEK2; PALB2 (2); PTEN; RAD50*
DCIS	26	11	42.3%	2	7.7%	2	7.7%	6	23.1%	38.5%	1	*PALB2*
ER+/no HER2 test	25	15	60.0%	2	8.0%	10	40.0%	1	4.0%	52.0%	2	*ATM; BRIP1*
No receptors	81	16	19.8%	5	6.2%	7	8.6%	2	2.5%	17.3%	2	*CHEK2 (2*)
Total	379	145	38.3%	75	19.8%	35	9.2%	22	5.8%	34.8%	13	
Grade/type												
Grade 1	12	1	8.3%	0	0.0%	1	8.3%	0	0.0%	8.3%		
Grade 2	48	15	31.3%	4	8.3%	8	16.7%	0	0.0%	25.0%	3	*ATM; BRIP1; PTEN*
Grade 3*	242	106	43.8%	65	26.9%	19	7.9%	14	5.8%	40.5%	8	*ATM; CHEK2 (2); PALB2 (3); RAD50; RECQL*
Lobular	8	3	37.5%	1	12.5%	1	12.5%	1	12.5%	37.5%		
DCIS	26	11	42.3%	2	7.7%	2	7.7%	6	23.1%	38.5%	1	*PALB2*
Unknown	43	9	20.9%	3	7.0%	4	9.3%	1	2.3%	18.6%	1	*CHEK2*
Total	379	145	38.3%	75	19.8%	35	9.2%	22	5.8%	34.8%	13	
Manchester Scoring System												
<15	106	14	13.2%	1	0.9%	2	1.9%	5	4.7%	7.5%	6	*CHEK2 (2); PALB2 (2); PTEN; RECQL*
15–19	119	28	23.5%	9	7.6%	7	5.9%	10	8.4%	21.8%	2	*BRIP1; PALB2*
20–24	64	29	45.3%	13	20.3%	10	15.6%	3	4.7%	40.6%	3	*ATM; CHEK2; PALB2*
25–39*	59	43	72.9%	29	49.2%	9	15.3%	3	5.1%	69.5%	2	*ATM; RAD50*
40>	31	31	100.0%	23	74.2%	7	22.6%	1	3.2%	100.0%		
Total	379	145	38.3%	75	19.8%	35	9.2%	22	5.8%	34.8%	13	
Bilateral breast cancer												
Bilateral	63	36	57.1%	18	28.6%	8	12.7%	10	15.9%	57.1%	1	*BRIP1*

*One case is *BRCA1* and *BRCA2* positive.

†Two cases are ER-/ HER2-, but PR+.

TNBC, triple-negative breast cancer.

The *CHEK2* c.1100delC PV was identified in only 2/379 (0.53%) compared with 1.7% (55/3177) in women with breast cancer aged >30 years (p=0.0835) seen at the MCGM and 2.3% in the POSH study aged ≤40% and 1% in POSH cases≤30 years (table 1).

### Manchester score

The detection of PVs in *BRCA1* and *BRCA2* was, as expected, strongly correlated with breast cancer pathology and family history. The MS accurately predicted the likelihood of a *BRCA1*/*BRCA2* variant at both the 10% (15–19 points) and 20% (20–24 points) thresholds (table 2). By including PVs in *TP53,* 100% of women with a MS ≥40 had a PV in *BRCA1/2* or *TP53*.

### Tumour characteristics

We identified 61 (48.8%) PVs in *BRCA1*/*2*/*TP53* in 125 women with triple-negative breast cancer (TNBC) (table 3). Unexpectedly, a similar rate of *BRCA1*/*2*/*TP53* PVs was detected in cases of pure DCIS (11/26 [42.3%]), although *TP53* accounted for 54.5% (6/11) of these. Eight were comedo DCIS of which four had a *TP53* PV. The majority of DCIS were high grade (18/26) and 8/18 harboured a PV (2 in *BRCA1*, 1 in *BRCA2* and 5 in *TP53*) (table 3). None of the cases of pure DCIS were detected on screening for familial risk.

**Table 3:** Rates of pathogenic variants found in patients with DCIS

DCIS	Total cases	Total PVs	%	*BRCA1*	%	*BRCA2*	%	*TP53*	%	*BRCA1/BRCA2/* *TP53* combined (%)	*PALB2*	%
Total	26	11	42.3	2	7.7	2	7.7	6	23.10	38.5	1	3.8
≤25 years	8	3	37.5	0	0.0	1	12.5	2	25.00	37.5	0	0.0
26–30 years	18	8	44.4	2	11.1	1	5.6	4	22.20	38.9	1	5.6
Grade/type												
Unknown	3	0	0.0	0	0.0	0	0.0	0	0	0.0	0	0.0
2	8	2	25.0	0	0.0	1	12.5	0	0%	12.5	1	12.5
3	14	8	57.1	2	14.3	1	7.1	5	36	57.1	0	0.0
Pagets	1	1	100.0	0	0.0	0	0.0	1	100	100.0	0	0.0
Comedo	8	4	50.0	0	0.0	0	0.0	4	50	50.0	0	0.0

PV, pathogenic variant.

HER2+ breast cancer showed a similar predominance of *TP53* PVs (8/43 (18.6%)), but *BRCA1/2* PVs were uncommon (3/43 (6.9%)).

Presence of cancer in both breasts was also predictive of PVs, with 36/63 (57.1%) cases with *BRCA1/2/TP53* PVs (including 10/22 *TP53* PVs) having bilateral breast cancer.

### Sporadic breast cancer

Of 147 women without a family history of breast or ovarian cancer at original diagnosis, 24 (16.3%) had a PV. Only 10 (6.8%) had *BRCA1/2* PVs (*BRCA1=*7; *BRCA2=*4; 1 woman had both *BRCA1* and *BRCA2* PVs), 12 women had a *TP53* PV and the remaining 2 women had a *PALB2* or a *CHEK2* PV. All *BRCA1* PVs were detected in women with sporadic TNBC 7/59 (11.9%). There were six other PVs identified in sporadic TNBC in *BRCA2*=3, *TP53*=2 and *PALB2*=1. Of 26 people with HER2+ sporadic breast cancers, 7 (26.9%) had PVs; (*TP53*=6; *BRCA2*=1). Outside of these confirmed pathologies 5/62 (8.1%) had PVs (*TP53*=4, *CHEK2*=1), but receptor status was unknown in 43 cases, including 13 with DCIS, two of whom had a *TP53* PV.

### *TP53* carriers

Among *TP53* carriers, 10/22 (45.5%) had a family history of breast cancer at initial diagnosis. Additional relatives in three of these families had Li Fraumeni spectrum tumours (one had none at diagnosis) and one had a personal history of childhood adrenocortical cancer. Additionally, four families without relatives with breast cancer, had family histories, including the index breast cancer, consistent with classical Li Fraumeni syndrome including at least one sarcoma aged <45 years. One *de novo* case had an osteosarcoma of the leg aged 19 years. Seven (33%) apparently *de novo* TP53-associated cases (confirmed after parental testing), with no significant personal or family history of cancer, presented with breast cancer. Thus, 7/144 (4.9%) apparently sporadic breast cancer cases ≤30 years had *TP53 de novo* variants that would not have been expected from personal or family history.

One of the *TP53* PVs was identified at a variant allele frequency of 22% suggesting mosaicism (online supplemental table 1). The PV was found in the tumour (20%-neoplastic content) at 15% and 11% in normal breast excluding clonal haematopoiesis (in a woman with Paget’s/DCIS who had not undergone radiotherapy/chemotherapy).

### Assessment of population level of testing

There were 135 women diagnosed with breast cancer in the Manchester region aged ≤30 years between 01/01/1990 and 31/12/1997 (since cancer genetic testing was introduced in Manchester) within the population study giving an annual rate of 16.9 cases. During this time, we tested 73/135 (54.1%) of affected women and identified *BRCA1=*13 (17.8%), *BRCA2=*8 (11%) and *TP53=*3 (4.1%) PVs. Of our population based study group of 125 women who underwent genetic testing (presenting with cancer between 1980 and 1997), there were PVs in *BRCA1=*23 (18.4%), *BRCA2*=11 (8.8%), *TP53*=5 (4%) and *BRIP1*=1,^12 13^ demonstrating a very similar overall detection rate. In the cohort referred to MCGM between 01/01/1998 and 3/11/2019, we tested 219 women and identified PVs in *BRCA1=*46 (21.0%), *BRCA2*=17 (7.8%) and *TP53*=16 (7.3%). The combined rate of *BRCA1/2* PVs at 27.2% (population-based study) and 28.8% (referrals) are similar, suggesting no substantial testing bias. However, 68/125 (54.4%) in the population study (1980–1997) had no family history, compared with 77/219 (35.2%) in the recent cases (1998–2019) (p=0.0006). All but 18 of the 219 tested since 1997 had full pathology and ER receptor status available, and only eight ER+ ductal carcinomas had unknown HER2 status.

### Co-occurrence of actionable breast cancer gene variants

Of 920 breast cancer cases with no prescreening tested at MCGM, no co-occurrence of two actionable breast cancer gene variants was found. Among 4916 non-Jewish breast cancer cases undergoing full *BRCA1* and *BRCA2* testing, only two co-occurrences of *BRCA1* and *BRCA2* PVs has occurred including the single case reported in this study.

## Discussion

We report here the results of 379 patients with breast cancer ≤30 years initially tested for PVs in *BRCA1, BRCA2, TP53* and *CHEK2* c.1100delC. Of the patients testing negative for these genes, 184 underwent testing of a panel of breast cancer associated genes. A total of 145 PVs were detected in 144 women, of which the majority (134 PVs) were identified in *BRCA1, BRCA2, TP53* and *CHEK2* c.1100delC. Only eight actionable PVs were found through extended panel testing. The rate of PVs in the unselected population series (n=125) was 18.9% in *BRCA1*, 8.8% in *BRCA2* and 4% in *TP53*. The overall detection rate for *TP53* (5.8%) in all samples is similar to the rate (6%) published previously.^17^ The Myriad study assessed this age group (783 women) and found combined rates of *BRCA1/2* PVs of 14% in women aged 25–29 years and 9% in women aged <25 years,^6^ although this cannot be considered a population study. Our study supports this lower detection rate in the very youngest age group, in contrast to the overall trend to increasing frequency of *BRCA1/2* at younger ages seen in population based testing.^21^ This is similar to the lower rates found in ovarian cancer <30 years.^22^ The Myriad study^6^ also showed a similarly increased detection rate for TNBC <30 years. Although there was no breakdown between *BRCA1* and *BRCA2*, it is highly likely that this was *BRCA1* driven as in our study. There is no specific figure given for *TP53* in this age group, but it is also likely that the increased detection rates for non-*BRCA* genes from <4% (similar to all other age groups) in the 25–29 age group to ~8% in the <25 group is due to *TP53*. In this study, we noted an increased detection rate from 4.8% to 11.7%, due to the inclusion of *TP53*. Specific data from 287 of the POSH cases diagnosed aged <31 who have been analysed for *TP53* and *CHEK2* c.1100delC in addition to *BRCA1/2* showed overall PV rate was higher in the <26 age group (28.9%) compared with 18.1% in the higher age group (online supplemental table 2). *TP53* and *BRCA2* PVs were more prevalent in the youngest age groups in the POSH study although numbers were small. Nonetheless, combining the frequencies from both studies the rates of *BRCA1* and *BRCA2* fell from 17.1% and 7.9% in the 26–30 age group to 10.1% and 7.1% in the <26 age group, respectively, although this was not significant for *BRCA1* (p=0.1) and combined *BRCA1* and *BRCA2* (p=0.09). The increase for *TP53* detection remained significant from 3.2% to 9.1% (p=0.01). The difference in incidence of PVs between POSH and this study may be due to sampling, certainly excluding cases with no invasive component to the presenting cancer would explain the lower rate of *TP53* in the POSH study as well as excluding previous malignancy which jointly made up 12/22 (54%) of *TP53* carriers in Manchester.

We have also analysed available online data from Ambry genetics commercial testing (https://www.ambrygen.com/providers/resources/prevalence-tool, accessed 29/08/2020).^23^ While it is not possible to assess the level of pretesting for *TP53*, and *BRCA1/2* or the presence of a Li Fraumeni family history, there is a clear upward trend of prevalence of *BRCA1* and *BRCA2* PVs with reducing age at breast cancer until 26 years of age (online supplemental table 3). In contrast *TP53* detection is increased in the <26 year age group (p=0.03), consistent with our findings.

Although the Myriad study is larger than the present study, there is a lack of detail, in particular regarding how much pretesting had been undertaken for PVs in *BRCA1/2/TP53*. Many women may have been tested for *BRCA1/2* years earlier and subsequently taken advantage of extended testing. Similarly, women diagnosed with breast cancer and features of Li Fraumeni syndrome may have undergone clinical bespoke *TP53* testing. Nine of 15 (60%) such *TP53* cases in the present study triggered clinical testing based on personal or family history. The lower rates for *BRCA1/2/TP53* PVs in the Myriad study probably reflects this level of pretesting and the more likely accurate rates are from the pure population-based series in the present study from 1980 to 1997.^16^

The current study has convincingly shown that PVs in *BRCA1* are the biggest contributor to breast cancer in women diagnosed aged ≤30 years. Even in the pure population-based study, this was at least twice the rate of *BRCA2. BRCA1* PVs were also twice as prevalent in this age group as *BRCA2* PVs in the POSH study. Given the lower population prevalence of *BRCA1* PVs, the risk of breast cancer in some women with a *BRCA1* PV will be sufficient to recommend MRI screening in *BRCA1* PV carriers<30 years. New UK guidance from the National Screening Committee will allow screening in *BRCA1/2* PV carriers once their 10 year risk is 8%.^24^ This level of risk is estimated in *BRCA1* PV carriers aged 25 years with a first degree relative diagnosed <40 years in both the Tyrer-Cuzick and BOADICEA models.^25 26^ Many other countries already offer screening in *BRCA1/2* PV carriers from 25 years. The presence of seven *TP53* carriers with breast cancer <26 years of age may well justify MRI screening from age 20 years as is already recommended in a number of guidelines.^24^

The present study has shown limited clinical benefit from testing of genes apart from *BRCA1*, *BRCA2* and *TP53* in women with invasive or *in situ* breast cancer aged ≤30 years. The individual with a *PTEN* PV had a classical phenotype and had *PTEN* bespoke testing rather than a panel. The detection rate in other actionable breast cancer genes was only 4.3% (8/184). Even allowing for an increased detection rate from testing the remaining 62 cases, this would have only reached 11/246 cases. Nevertheless, as at least seven *TP53* cases would not have been suspected based on personal or family history, *TP53* should be included in first-line testing as long as the panel does not reduce sensitivity for *BRCA1*/*2* variant detection. While a single *BRIP1* PV was detected, this gene is not convincingly associated with breast cancer risk and the current evidence does not support actionability for these variants.^27^ Similarly there has been no clinical validation for *RECQL* 28 29 and *RAD50* and the cases in the current series was consistent with population frequencies. We also found no *RAD51C* or *RAD51D* variants consistent with their primary association with ovarian cancer susceptibility.^30 31^

All different tumour pathologies had a >9% detection rate for *BRCA1*/*2* and *TP53* PVs. A striking finding was that the rate of PVs associated with DCIS (42.3%) was almost as high as that associated with TNBC (48.3%). The previous association with *TP53* and high-grade comedo DCIS was noted.^13^ We also found a rate of 15.4% (4/26) for *BRCA1/2* PVs in DCIS cases. The 23.1% rate for *TP53* PVs in DCIS in our study reflects the very strong association of DCIS even with invasive cancers with 41 of 45 (91.1%) of all cases containing DCIS in one study of *TP53* related breast cancers.^32^ Currently, many countries in Europe have not instituted extended panel testing for breast cancer and in England testing for a three gene panel of *BRCA1*, *BRCA2* and *PALB2* will be provided by the public healthcare system unless a specific request is made for *TP53* by a geneticist. Our study would suggest that *TP53* should be discussed and potentially added to all breast cancer gene screens≤30 years unless the woman declines following counselling of the implications of this test. The importance of identifying *TP53* variants is shown by the extremely high rate of contralateral breast cancer, nearly 50% in the present study and with annual contralateral rates of ~40%.^33^ Given the concerns about radiation treatment and new primaries with *TP53*,^34 35^ a discussion about mastectomy and even bilateral mastectomy needs to be undertaken as well as instituting proven early detection strategies for other malignancies, including whole body MRI as published in two recent guidelines.^34 35^

This study has some limitations. Not all 379 women underwent full testing of the panel of breast cancer associated genes. However, we have shown that there is a very low likelihood that an individual identified with a PV in *BRCA1/2* or *TP53* would also carry a PV in another breast cancer gene. It is therefore unlikely that failure to test those with known *BRCA1/2* PVs missed PVs in other breast cancer genes. Unfortunately, full pathology and receptor status was not available on all women. This reflects the chronological, real life data nature of the study. Breast cancer grade was only reported reliably after 1990 and ER receptor status after 1995. HER2 status was not usually reported until 1999, after approval of Herceptin (trastuzumab) for treating HER2+ breast cancer. Nonetheless, there were still a large number of TNBCs available for assessment and since 1997 the majority of women had full pathology available, including HER2 status. The strengths of this study include: the large number of patients with what is a rare cancer in young women; the well characterised nature of the cohort with extensive family history; a pure population-based cohort with high ascertainment even in the postcohort study period, and the presence of a population control for evaluated genes. The sensitivity of our testing, especially for *BRCA1*/*2* and *TP53*, is high, indicated by the 100% detection rate of a PV in the 31 women with MS of ≥40. Although the score was designed for *BRCA1/2*, it has also clearly captured very early onset highly penetrant *TP53* families.

In conclusion, we have identified a high rate of actionable PVs in breast cancer genes in women with breast cancer aged ≤30 years. The clear association of *TP53* PVs in very young women presenting only with DCIS is noteworthy and adds to the published association of HER2+ invasive disease in young women with *TP53* PVs.^32^ *TP53* and *BRCA1/2* PVs are of similar frequency in women with breast cancer <26 years but *BRCA1/2* PVs predominate in those aged 26–30 years. Overall, there is little additional benefit of testing breast cancer-associated genes apart from *BRCA1*, *BRCA2* and *TP53* in this age group.

## Data Availability

Data are available on reasonable request. The datasets analysed during the current study are available from the corresponding author on reasonable request.
