# Supplementary material for: High likelihood of actionable pathogenic variant detection in breast cancer genes in women with very early onset breast cancer
Source: J Med Genet. 2021 Mar 23;59(2):115–21. doi: 10.1136/jmedgenet-2020-107347 (PMC8788257; doi:10.1136/jmedgenet-2020-107347)
Supplement: Supplementary data [file jmedgenet-2020-107347supp001.pdf]

Supplementary Table 1: Pathogenic variants identified in women diagnosed with breast cancer at the age of thirty or younger.

Nr	MS	Gene	Variant	Type variant	ACMG	Age group	DCIS/ Lobular/ Invasive	Grade	Receptor Status	Sporadic breast cancer	Bilateral breast cancer
1	36	*ATM*	c.2165dupT; p.(Leu722Phefs*16)	Truncating	5	<26	Invasive	2	ER+	No	
2	24	*BRCA1*	c.34C>T; p.(Gln12*)	Truncating	5	<26	Invasive	3	ER-/HER2-	No	
3	24	*BRCA1*	c.4065_4068delTCAA; p.(Asn1355Lysfs*10)	Truncating	5	<26	Invasive	3	ER+/HER2-	No	Yes
4	25	*BRCA1*	c.4065_4068delTCAA; p.(Asn1355Lysfs*10)	Truncating	5	<26	Invasive	3	ER-/HER2-	No	
5	43	*BRCA1*	c.547+1G>T; p.?	Truncating	5	<26	Invasive	3	ER-/HER2-	No	
6	27	*BRCA1*	c.3779delT; p.(Leu1260Tyrfs*4)	Truncating	5	<26	Invasive	3	ER-/HER2-	No	
7	39	*BRCA1*	c.3668_3671dup; p.(Cys1225Serfs*10)	Truncating	5	<26	Invasive	3	ER-/HER2-	No	
8	20	*BRCA2*	c.3599_3600delGT; p.(Cys1200*)	Truncating	5	<26	Invasive	3	ER-/HER2-	Yes	
9	23	*BRCA2*	c.7757G>A; p.(Trp2586*)	Truncating	5	<26	Invasive	3	ER+	No	
10	34	*BRCA2*	c.5946delT; p.(Ser1982Argfs*22)	Truncating	5	<26	DCIS			No	Yes
11	25	*TP53*	c.571_572delCC; p.(Pro191Serfs*17)	Truncating	5	<26	DCIS			No	
12	55	*TP53*	c.993+1G>A; p.?	Splice site	5	<26	DCIS			No	Yes
13	6	*TP53*	c.524G>A; p.(Arg175His)	Missense	5	<26	Invasive	3	ER+/HER2+	Yes	
14	17	*TP53*	c.949C>T; p.(Gln317*)	Truncating	5	<26	Lobular		ER+/HER2+	Yes	Yes
15	24	*TP53*	c.742C>T; p.(Arg248Trp)	Missense	5	<26	Invasive	3	ER+/HER2-	No	
16	18	*TP53*	c.730G>A; p.(Gly244Ser)	Missense	5	<26	Invasive	3		Yes	Yes
17	6	*TP53*	c.638G>A; p.(Arg213Gln)	Missense	5	<26	Invasive	3	ER+/HER2+	Yes	
18	20	*ATM*	c.3802delG; p.(Val1268*)	Truncating	5	26-30	Invasive	3	ER+/HER2-	No	
19	24	*BRCA1*	c.1953dupG; p.(Lys652Glufs*21)	Truncating	5	26-30	DCIS			No	
20	47	*BRCA1*	c.5362G>A; p.(Gly1749Asp)	Missense	4	26-30	Invasive	3	ER-/HER2-	No	
21	32	*BRCA1*	c.5194-12G>A; p.?	Splice site	4	26-30	Invasive	3	ER-/HER2-	No	
22	32	*BRCA1*	c.68_69delAG; p.(Glu23Valfs*17)	Truncating	5	26-30	Invasive	3	ER-/HER2-	No	
23	34	*BRCA1*	c.5503C>T; p.(Arg1835*)	Truncating	5	26-30	Invasive	3	ER-/HER2-	No	
24	41	*BRCA1*	c.3358_3359delGT; p.Val1120*	Truncating	5	26-30	Invasive	3	ER-/HER2-	No	

25	40	*BRCA1*	c.4438dupT; p.(Ser1480Phefs*4)	Truncating	5	26-30	Invasive	3	ER-/HER2-	No	Yes
26	39	*BRCA1*	c.1961dupA; p.(Tyr655Valfs*18)	Truncating	5	26-30	Invasive	3	ER+	No	
27	55	*BRCA1*	c.4065_4068delTCAA; p.(Asn1355Lysfs*10)	Truncating	5	26-30	Invasive	3	ER-/HER2-	No	Yes
28	35	*BRCA1*	c.2563C>T; p.Gln855*	Truncating	5	26-30	Invasive	3	ER-/HER2-	No	
29	43	*BRCA1*	c.68_69delAG; p.(Glu23Valfs*17)	Truncating	5	26-30	Lobular		ER+	No	Yes
30	25	*BRCA1*	c.68_69delAG; p.(Glu23Valfs*17)	Truncating	5	26-30	Invasive	3	ER-/HER2-	Yes	Yes
31	23	*BRCA1*	c.427G>T; p.(Glu143*)	Truncating	5	26-30	Invasive	3	ER-/HER2-	No	
32	23	*BRCA1*	Deletion exons 1-17	CNV	5	26-30	Invasive	3	ER-/HER2-	No	
33	19	*BRCA1*	Deletion exons 1-2	CNV	5	26-30	NOS			No	
34	54	*BRCA1*	c.302-2delA; p.?	Splice site	5	26-30	DCIS			No	
35	37	*BRCA1*	Deletion exons 15-16	CNV	5	26-30	Invasive	3	ER-/HER2-	No	Yes
36	35	*BRCA1*	c.-107A>T; p.?	Promotor	5	26-30	Invasive	3	ER-/HER2-	No	Yes
37	59	*BRCA1*	c.1757delC; p.(Pro586Leufs*2)	Truncating	5	26-30	Invasive	3	ER-/HER2-	No	
38	25	*BRCA1*	Duplication exons 18-19	CNV	5	26-30	Invasive	3	ER-/HER2-	No	Yes
39	24	*BRCA1*	c.3396delC; p.(Leu1133*)	Truncating	5	26-30	Invasive	2	ER+/HER2-	No	
40	30	*BRCA1*	c.5117G>A; p.(Gly1706Glu)	Missense	5	26-30	Invasive	3	ER-/HER2-	No	Yes
41	38	*BRCA1*	Deletion exons 1-2	CNV	5	26-30	Invasive	3	ER-/HER2-	No	
42	14	*BRCA1*	Duplication exon 13	CNV	5	26-30	Invasive	3	ER-/HER2-	Yes	
43	45	*BRCA1*	c.3607C>T; p.(Arg1203*)	Truncating	5	26-30	Invasive	3	ER-/HER2-	No	
44	17	*BRCA1*	Duplication exon 13	CNV	5	26-30	Invasive	3	ER-/HER2-	Yes	
45	71	*BRCA1*	Deletion exon 24	CNV	5	26-30	Invasive	3	ER-/HER2-	No	
46	15	*BRCA1*	c.1961dupA; p.(Tyr655Valfs*18)	Truncating	5	26-30	Invasive	3	ER+/HER2-	No	
47	47	*BRCA1*	c.3598C>T; p.(Gln1200*)	Truncating	5	26-30	Invasive	3	ER-/HER2-*	No	
48	17	*BRCA1*	c.4065_4068delTCAA; p.(Asn1355Lysfs*10)	Truncating	5	26-30	Invasive	3	ER-/HER2-	Yes	
49	17	*BRCA1*	c.4625_4626delCT; p.(Ser1542Trpfs*31)	Truncating	5	26-30	Invasive	3	ER-/HER2-	Yes	
50	28	*BRCA1*	c.3400G>T; p.(Glu1134*)	Truncating	5	26-30	Invasive	3		No	

51	36	*BRCA1*	c.1175_1214del; p.(Leu392Glnfs*5)	Truncating	5	26-30	Invasive	3	ER-/HER2-	No	
52	31	*BRCA1*	c.5108A>G; p.(Tyr1703Cys)	Missense	4	26-30	Invasive	3	ER-/HER2-	No	
53	31	*BRCA1*	c.4065_4068delTCAA; p.(Asn1355Lysfs*10)	Truncating	5	26-30	Invasive	3	ER+/HER2-	No	Yes
54	25	*BRCA1*	c.5179A>T; p.(Lysl727*)	Truncating	5	26-30	Invasive	3	ER-/HER2-	No	
55	18	*BRCA1*	c.4065_4068delTCAA; p.(Asn1355Lysfs*10)	Truncating	5	26-30	Invasive	3	ER-/HER2-	No	
56	22	*BRCA1*	c.3331_3334delCAAG; p.(Gln1111Asnfs*5)	Truncating	5	26-30	Invasive	3	ER+/HER2-	No	
57	44	*BRCA1*	c.3869_3870del; p.(Lys1290Metfs*4)	Truncating	5	26-30	Invasive	3	ER-/HER2-	No	
58	34	*BRCA1*	c.63_64delinsA; p.(Leu22*)	Truncating	5	26-30	Invasive	3		No	Yes
59	28	*BRCA1*	c.2475delC; p.(Asp825Glufs*21)	Truncating	5	26-30	Invasive	3	ER+/HER2-	No	Yes
60	18	*BRCA1*	c.5503C>T; p.(Arg1835*)	Truncating	5	26-30	Invasive	3	ER+/HER2-	No	
61	21	*BRCA1*	c.212+1G>T; p.?	Splice site	5	26-30	NOS			No	
62	29	*BRCA1*	c.4065_4068delTCAA; p.(Asn1355Lysfs*10)	Truncating	5	26-30	Invasive	3	ER-/HER2-	No	
63	15	*BRCA1*	c.3598C>T; p.(Gln1200*)	Truncating	5	26-30	Invasive	3	ER+/HER2+	No	
64	31	*BRCA1*	c.213-11T>G; p.?	Splice site	5	26-30	Invasive	3	ER-/HER2-	No	Yes
65	31	*BRCA1*	Duplication exon 13	CNV	5	26-30	Invasive	3	ER-/HER2-	No	
66	17	*BRCA1*	c.38delA; p.(Asn13Metfs*10)	Truncating	5	26-30	Invasive	3	ER-/HER2-	Yes	
67	21	*BRCA1*	c.4123G>T; p.(Glu1375*)	Truncating	5	26-30	Invasive	3	ER+/HER2-	No	Yes
68	21	*BRCA1*	Deletion exons 21-24	CNV	5	26-30	Invasive	3	ER+/HER2-	No	
69	31	*BRCA1*	Deletion exons 1-21	CNV	5	26-30	NOS			No	Yes
70	30	*BRCA1*	c.2681_2682delAA; p.(Lys894Thrfs*8)	Truncating	5	26-30	Invasive	3	ER-/HER2-	No	
71	20	*BRCA1*	c.1423_1508del; p.(Ser475Alafs*2)	Truncating	5	26-30	Invasive	3	ER-/HER2-	No	
72	50	*BRCA1*	c.5503C>T; p.(Arg1835*)	Truncating	5	26-30	Invasive	3	ER-/HER2-	No	
73	56	*BRCA1*	c.5152+4A>G; p.?	Splice site	4	26-30	Invasive	3	ER+/HER2-	No	
74	45	*BRCA1*	c.427G>T; p.(Glu143*)	Truncating	5	26-30	Invasive	2	ER+/HER2-	No	
75	52	*BRCA1*	c.5387dupC; p.(Ser1797Ilefs*33)	Truncating	5	26-30	Invasive	3	ER-/HER2-	No	Yes
76	53	*BRCA1*	c.5362G>A; p.(Gly1749Asp)	Missense	4	26-30	Invasive	3	ER+/HER2-	No	

77	45	*BRCA1*	c.5406+1G>A; p.?	Splice site	5	26-30	Invasive	3	ER-/HER2-	No	
78	48	*BRCA1*	c.3869_3870del; p.(Lys1290Metfs*4)	Truncating	5	26-30	Invasive	3	ER-/HER2-	No	
79	42	*BRCA1*	Duplication exons 18-19	CNV	5	26-30	Invasive	3	ER-/HER2-	No	
80	50	*BRCA1*	c.4065_4068delTCAA; p.(Asn1355Lysfs*10)	Truncating	5	26-30	Invasive	3	ER-/HER2-	No	
81	64	*BRCA1*	c.4327C>T; p.(Arg1443*)	Truncating	5	26-30	Invasive	2	ER-/HER2-	No	
82	48	*BRCA1*	c.1961delA; p.(Lys654Serfs*47)	Truncating	5	26-30	Invasive	3	ER+/HER2-	No	Yes
83	30	*BRCA1*	c.2071delA; p.(Arg691Aspfs*10)	Truncating	5	26-30	Invasive	3	ER+/HER2-	No	
84	27	*BRCA1;*<br>*BRCA2*	c.4625_4626delCT; p.(Ser1542Trpfs*31);<br>c.5073dupA; p.(Trp1692Metfs*3)	Truncating	5<br>5	26-30	Invasive	3	ER-/HER2-	Yes	Yes
85	24	*BRCA2*	c.1929delG; p.(Arg645Glufs*15)	Truncating	5	26-30	NOS			No	
86	23	*BRCA2*	c.6275_6276del; p.(Leu2092Profs*7)	Truncating	5	26-30	Invasive	3	ER+	No	
87	34	*BRCA2*	Deletion exon 2	CNV	5	26-30	Invasive	2	ER+	No	Yes
88	18	*BRCA2*	c.6275_6276del; p.(Leu2092Profs*7)	Truncating	5	26-30	Invasive	3	ER+	No	
89	14	*BRCA2*	c.7480C>T; p.(Arg2494*)	Truncating	5	26-30	Invasive	3		No	
90	25	*BRCA2*	c.1058C>A; p.(Ser353*)	Truncating	5	26-30	Invasive	2		No	
91	16	*BRCA2*	c.8904delC; p.(Val2969Cysfs*7)	Truncating	5	26-30	Invasive	3	ER+	No	
92	22	*BRCA2*	c.574_575delAT; p.(Met192Valfs*13)	Truncating	5	26-30	Invasive	3	ER+	No	
93	102	*BRCA2*	c.6591_6592delTG; p.(Glu2198Asnfs*4)	Truncating	5	26-30	Invasive	3	ER+	No	
94	21	*BRCA2*	c.1929delG; p.(Arg645Glufs*15)	Truncating	5	26-30	Invasive	2	ER+	No	
95	18	*BRCA2*	c.7988A>T; p.(Glu2663Val)	Missense	5	26-30	Invasive	3	ER-/HER2+	No	
96	17	*BRCA2*	c.4478_4481delAAAG; p.(Glu1493Valfs*10)	Truncating	5	26-30	Invasive	3	ER+	No	
97	44	*BRCA2*	c.8912delA; p.(Lys2971Serfs*5)	Truncating	5	26-30	Invasive	3	ER+/HER2-	No	
98	46	*BRCA2*	c.5682C>G; p.(Tyr1894*)	Truncating	5	26-30	NOS			No	Yes
99	11	*BRCA2*	c.5073dupA; p.(Trp1692Metfs*3)	Truncating	5	26-30	Invasive	3	ER+/HER2+	Yes	Yes
100	29	*BRCA2*	c.9294C>G; p.Tyr3098*	Truncating	5	26-30	NOS			No	Yes
101	43	*BRCA2*	c.26delC; p.(Pro9Glnfs*16)	Truncating	5	26-30	Invasive	3	ER-/HER2-	No	Yes

102	23	*BRCA2*	c.7069_7070delCT; p.(Leu2357Valfs*2)	Truncating	5	26-30	Invasive	3	ER-/HER2-	No	
103	42	*BRCA2*	c.1929delG; p.(Arg645Glufs*15)	Truncating	5	26-30	Invasive	2		No	
104	16	*BRCA2*	c.5303_5304delTT; p.(Leu1768Argfs*5)	Truncating	5	26-30	Lobular		ER+/HER2-	No	
105	18	*BRCA2*	c.1813delA; p.(Ile605Tyrfs*9)	Truncating	5	26-30	Invasive	3	ER-/HER2-	Yes	
106	24	*BRCA2*	c.2330dupA; p.(Asp777Glufs*11)	Truncating	5	26-30	Invasive	3	ER+/HER2-	No	
107	23	*BRCA2*	c.1189_1190insTTAG; p.(Gln397Leufs*25)	Truncating	5	26-30	NOS			No	
108	16	*BRCA2*	c.2899_2900delCT; p.(Leu967Argfs*14)	Truncating	5	26-30	Invasive	2	ER+	No	
109	27	*BRCA2*	c.1964delC; p.(Pro655Glnfs*5)	Truncating	5	26-30	Invasive	2	ER+/HER2-	No	
110	29	*BRCA2*	c.7024C>T; p.(Gln2342*)	Truncating	5	26-30	DCIS			No	
111	107	*BRCA2*	c.6591_6592delTG; p.(Glu2198Asnfs*4)	Truncating	5	26-30	Invasive	3	ER-/HER2-	No	Yes
112	41	*BRCA2*	Deletion exons 1-24	CNV	5	26-30	Invasive	2	ER+/HER2-	No	
113	25	*BRCA2*	c.2808_2811delACAA p.(Ala938Profs*21)	Truncating	5	26-30	Invasive	2	ER+/HER2-	No	
114	21	*BRCA2*	c.5073dupA; p.(Trp1692Metfs*3)	Truncating	5	26-30	Invasive	1	ER+/HER2-	No	
115	28	*BRCA2*	c.4391_4392delCT; p.(Ser1464*)	Truncating	5	26-30	Invasive	3	ER+/HER2-	No	
116	19	*BRIP1*	c.2392C>T; p.(Arg798*)	Truncating	5	26-30	Invasive	2	ER+	No	Yes
117	11	*CHEK2*	c.1100delC; p.(Thr367Metfs*15)	Truncating	5	26-30	NOS			Yes	
118	13	*CHEK2*	c.1100delC; p.(Thr367Metfs*15)	Truncating	5	26-30	Invasive	3		No	
119	24	*CHEK2*	c.283C>T; p.(Arg95*)	Truncating	5	26-30	Invasive	3	ER+/HER2-	No	
120	17	*PALB2*	Translocation	CNV	5	26-30	Invasive	3	ER-/HER2-	Yes	
121	12	*PALB2*	c.3113G>A; p.(Trp1038*)	Truncating	5	26-30	DCIS			No	
122	20	*PALB2*	c.3113G>A; p.(Trp1038*)	Truncating	5	26-30	Invasive	3	ER+/HER2-	No	
123	14	*PTEN*	c.253+1G>A; p.?	Splice site	5	26-30	Invasive	2	ER+/HER2-	No	
124	25	*RAD50*	c.1300_1306del; p.(Asp434Lysfs*7)	Truncating	5	26-30	Invasive	3	ER+/HER2-	No	
125	14	*RECQL*	c.1667_1667+3delAGTA; p.(Glu557Lysfs*14)	Truncating	5	26-30	Invasive	3	ER+/HER2+	No	
126	17	*TP53*	c.818G>A; p.(Arg273His)	Missense	5	26-30	Invasive	3	ER-/HER2-	Yes	
127	28	*TP53*	c.659A>G; p.(Tyr220Cys)	Missense	5	26-30	Invasive	3	ER-/HER2+	Yes	

BMJ Publishing Group Limited (BMJ) disclaims all liability and responsibility arising from any reliance placed on this supplemental material which has been supplied by the author(s)

128	10	*TP53*	c.818G>A; p.(Arg273His)	Missense	5	26-30	DCIS			Yes	Yes
129	8	*TP53*	c.574C>T; p.(Gln192*)	Truncating	5	26-30	DCIS			Yes	
130	28	*TP53*	c.659A>G; p.(Tyr220Cys)	Missense	5	26-30	Invasive	3	ER+/HER2+	Yes	Yes
131	21	*TP53*	c.375G>T; p.(Thr125Thr)	Splice site	5	26-30	Invasive	3	ER+	No	
132	19	*TP53*	c.1009C>T; p.(Arg337Cys)	Missense	5	26-30	Invasive	3	ER-/HER2+	No	Yes
133	19	*TP53*	c.743G>A; p.(Arg248Gln)	Missense	5	26-30	NOS			Yes	Yes
134	13	*TP53*	c.524G>A; p.(Arg175His) (22%)	Missense	5	26-30	DCIS			No	
135	19	*TP53*	c.919+1G>A; p.?	Splice site	5	26-30	DCIS			No	Yes
136	17	*TP53*	c.832C>T; p.(Pro278Ser)	Missense	4	26-30	Invasive	3	ER-/HER2-	No	
137	16	*TP53*	c.916C>T; p.(Arg306*)	Truncating	5	26-30	Invasive	3	ER-/HER2+	No	
138	17	*TP53*	c.916C>T; p.(Arg306*)	Truncating	5	26-30	Invasive	3	ER-/HER2-	Yes	
139	16	*TP53*	c.733G>A; p.(Gly245Ser)	Missense	5	26-30	Invasive	3	ER+/HER2+	Yes	Yes
140	20	*TP53*	c.896_909delTGCCCCCAGGGAGC; p.(Leu299Hisfs*2)	Truncating	5	26-30	Invasive	3	ER+/HER2-	No	Yes
141	12	*PALB2*	c.1467_1468delTC; p.(Pro490Argfs*5)	Truncating	5	<26	Invasive	3	ER+/HER2-	No	
142	23	*BRCA1*	c.191G>A; p(Lys64Tyr)	Missense	4	26-30	Invasive	3	ER-/HER2-	No	
143	24	*BRCA1*	Deletion exon 13	CNV	5	26-30	Invasive	2	ER-/HER2-	No	
144	27	*BRCA1*	c.1033_1039dup; p.(Leu347Argfs*5)	Truncating	5	26-30	Invasive	3	ER-/HER2-	No	

MS: Manchester score. ACMG: American college of Medical Genetics classification. CNV: copy number variant.

Supplementary Table 2: Rates of pathogenic variants found in the POSH study

POSH	Total cases	Total PVs	%	*BRCA1*	%	*BRCA2*	%	*TP53*	%	*BRCA1/BRCA2/ TP53* combined	*CHEK2 c.1100delC*	%
≤25 years	38	11	28.9%	4	10.5%	4	10.5%	2	5.3%	26.3%	1	2.63%
26-30 years*	249	45	18.1%	28	11.2%	13	5.2%	3	1.2%	17.7%	2	0.80%
Total	287	56	19.5%	32	11.1%	17	5.9%	5	1.7%	18.8%	3	1.05%

* One women carried both a *BRCA2* and *CHEK2* c.1100delC pathogenic variant.

Supplementary Table 3: Prevalence rate by age group from the Ambry Genetics online tool.

Age group	<26y	26-30y	31-40y	41-50y	51-60y	61-70y	>70y
***BRCA1* PV**	5%	5.77%	3.68%	1.40%	1.15%	0.81%	0.51%
***BRCA2* PV**	2.33%	3.29%	2.48%	1.56%	1.64%	1.58%	1.39%
***TP53* PV**	4.19%	1.93%	0.50%	0.18%	0.22%	0.15%	0.13%
***CHEK2* PV**	2.76%	3.50%	2.63%	2.75%	2.52%	2.64%	2.04%
***ATM* PV**	2.30%	2.07%	1.66%	1.15%	1.24%	0.98%	0.92%
***PALB2* PV**	2.62%	0.52%	1.03%	0.95%	1.05%	0.83%	0.49%
**Total individuals tested**	300	1247	10462	24016	14582	8401	3090

PV: pathogenic variant.
